# The Use of Tissue Grafts Associated with Immediate Implant Placement to Achieve Better Peri-Implant Stability and Efficacy: A Systematic Review and Meta-Analysis

**DOI:** 10.3390/jcm13030821

**Published:** 2024-01-31

**Authors:** Enrico Maria Rondone, Bruno Leitão-Almeida, Miguel Silva Pereira, Gustavo Vicentis Oliveira Fernandes, Tiago Borges

**Affiliations:** 1 Faculty of Dental Medicine, Universidade Católica Portuguesa, 3504-505 Viseu, Portugal; 2 Centre for Interdisciplinary Research in Health (CIIS), Universidade Católica Portuguesa, 3504-505 Viseu, Portugal; 3 A. T. Still University—Missouri School of Dentistry & Oral Health, St. Louis, MO 63104, USA

**Keywords:** dental implants, tissue grafting, tooth socket, systematic review, meta-analysis

## Abstract

**Background**: The goal of this systematic study and meta-analysis was to evaluate the efficacy of hard and/or soft tissue grafts associated with type-1 implants on healing and treatment outcomes. The primary outcomes studied were implant survival rate, pocket depth, marginal peri-implant recession, bone loss, bone thickness (volumetric change), interproximal bone level, mesial and distal papilla migration, and radiographic evaluation; and the secondary parameters were Pink Esthetic Score (PES), vertical distance from implant shoulder and bone, Visual Analogue Score (VAS), Implant Stability Quotient (ISQ), and biological complications (fistulas, pain, mucositis, and peri-implantitis). **Methods**: The PICO strategy was used to formulate the hypothesis under study: “For patients who underwent extraction and immediate implant placement, what is the efficacy of using any type of graft (bone or soft tissue) compared to non-grafting regarding the peri-implant parameters?” The electronic search process was performed on the MedLine/PubMed and Cochrane databases. It included randomized controlled trials (RCTs) from the last 11 years (from 2012 to November 2023), which were identified and analyzed. **Results**: Nine RCTs (κ = 0.98) were selected (403 patients and 425 implants); they were divided into three groups: bone graft (75 patients and 75 implants inserted), bone graft and membrane (213 patients and 235 implants inserted), and without bone graft (115 patients and 115 implants inserted). Three studies calculated the mid-facial mucosa level and two reported better results when a connective tissue graft was combined with the xenograft, whereas another study found better results in the combination of a dual-zone technique with a xenograft. Three studies evaluated the total Pink Esthetic Score (PES) at 12 months, where the authors found no significant difference in using a xenogeneic graft with or without a membrane. In the same period, the facial bone thickness was assessed in two articles; the authors reported better results in graft-treated and flapless groups. The risk-of-bias assessment found four studies with low risk, four with moderate risk, and one with a high risk of bias. The meta-analysis showed a medium level of heterogeneity for the mid-facial mucosa level analysis (I^2^ = 46%) and an overall effect size of 0.79 (95% CI [0.18; 1.40]), a statistically significant results (*p* = 0.01), with a tendency to favor the experimental group. Also, there was a medium level of heterogeneity among studies regarding total PES (I^2^ = 45%), with no significant differences between studies (*p* = 0.91). Homogeneous results (I^2^ = 0%) were found among studies analyzing facial bone thickness, favoring the experimental group; the forest plot showed an effect of 0.37 (95% CI [0.25; 0.50]), which was statistically significant (*p* < 0.00001) for this parameter. **Conclusions**: Then, it was possible to conclude that using bone and soft tissue grafting techniques associated with immediate implant placement (IIP), even though they are not fundamental, was a valuable resource to prevent significant tissue reduction, reaching greater bone stability and higher levels in the Pink Esthetic Score (PES) and Visual Analogue Score (VAS).

## 1. Introduction

Implant rehabilitation techniques involve using biomaterials [1,2] and titanium/zirconia to replace one or more teeth [3]. The classification of implant surgery techniques was developed based on alveolar healing times and included the following: type-1 protocol, immediate implantation (IIP), extraction and insertion in the same surgical protocol; type-2 protocol, early implant, insertion after 4–8 weeks after extraction, soft tissue healing; type-3 protocol, early-delayed implant, insertion after 12–16 weeks, partial healing of the alveolar bone component; and type-4 protocol, late implant, insertion after six months, complete healing of the alveolar bone component [4].

The type-1 protocol is a predictable treatment modality with success rates comparable to the type-4 technique [5,6,7,8]. This protocol brings several advantages, such as shorter procedures, reduced number of procedures, reduced vertical and horizontal resorption, and ideal gingival tissue esthetics [9,10,11]. The surgical criteria advocated for type-1 implants are an intact facial bone wall with a thick phenotype (greater than 1 mm), thick gingival biotype, absence of acute infection, and apical and palatal bone volume suitable for implant placement with sufficient primary stability [12].

As a result of post-extraction implant insertion into the socket, a gap is formed between the inner surface of the buccal cortical plate and the implant, named the jumping gap. Management of the gap is a critical decision for the clinician, who must choose to fill it with a graft or leave it clear with the blood clot alone [13]. In the 1990s, guided bone regeneration (GBR) was introduced, and alternatives were included, including using different grafts associated with a membrane placement [13]. The GBR technique has been increasingly indicated in type-1 (immediate implant) rehabilitation treatments claiming the purpose to compensate for volumetric changes in hard and soft tissues by using autogenous bone tissue grafts, deproteinized bovine bone mineral (DBBM) as fillers, and connective tissue grafts (CTG) and xenogeneic collagen grafts for post-placement alveolus closure [14].

Buser et al. [9] argued that the implant should be placed 2 mm from the inner surface of the buccal cortical plate to facilitate appropriate gap filling with the bone graft. Preclinical studies suggest that a smaller gap results in greater vertical resorption of the cortical bone [9]. The use of xenogeneic collagen material and a connective tissue graft to seal the socket and promote increased peri-implant keratinized mucosal volume is also well documented in the literature [15]. The xenogeneic resorbable matrix provides advantages such as faster healing and fewer surgeries since no surgical procedure is required to harvest the connective tissue graft [16,17]. In addition, DBBM placed in the marginal gap area reduces the amount of horizontal and vertical bone resorption associated with type-1 implant treatment [16].

It shows that a 4-walled defect is more favorable and presents reduced evidence of post-extraction ridge resorption because of the capability of containing the graft and greater effective capacity to incorporate the graft material [17]. Buser et al. [9] concluded that GBR surgical techniques are indeed effective in promoting bone filling and partial or complete resolution of cortical defects; they are more successful when associated with type-1 and -2 implants than late implants [12]. Therefore, there is a lack of uniformity about using or not tissue graft associated with IIP, and the type utilized if applicable. Thus, the objective of this systematic study was to review the literature in order to evaluate the efficacy of hard and/or soft tissue grafts associated with type-1 implants on healing and treatment outcomes. The null hypothesis was that the use of any graft material did not change or improve the healing process and esthetic result. The primary outcome variables were implant survival rate, pocket depth, marginal peri-implant recession, bone loss, bone thickness (volumetric change), interproximal bone level, mesial and distal papilla migration, and radiographic evaluation. The secondary parameters were Pink Esthetic Score (PES), vertical distance from implant shoulder and bone, Visual Analogue Score (VAS), Implant Stability Quotient (ISQ), and biological complications (fistulas, pain, mucositis, and peri-implantitis).

## 2. Materials and Methods

### 2.1. Protocol and PICO Strategy

The protocol of this systematic study was performed according to the PRISMA (Preferred Reporting Items for Systematic Reviews and Meta-Analyses) guidelines [18,19] and registered in the PROSPERO platform (International Prospective Register of Systematic Reviews, www.prospero.org, accessed on 10 January 2024; CRD42023383620).

The PICO (Population, Intervention, Comparison, and Outcome) strategy was used as the research model. The following question was posed to formulate the hypothesis under study: “For patients who underwent extraction and immediate implant placement, what is the efficacy of using any type of graft (bone or soft tissue) compared to non-grafting regarding the peri-implant parameters?” Population (P): Patients with a hopeless maxillary/mandibular tooth in the posterior or anterior areas who have received a type-1 implant with or without hard and soft tissue grafting; Intervention (I): Type-1 implant placement with or without hard and soft tissue grafting; Comparison (C): Hard and/or soft tissue grafting and standard healing; Outcome (O): Soft and hard tissue response as measured with the Pink Esthetic Score (PES), midfacial mucosa height, marginal bone loss (MBL), papilla index (PI), linear buccal change, volumetric change, bleeding on probing (BOP), and plaque index.

### 2.2. Eligibility Criteria

The inclusion criteria established for this review were as follows: randomized clinical trials (RCTs) that enrolled a minimum of 20 patients, had a follow-up of at least six months, and were published in the English language; studies that evaluated the efficacy of hard and/or soft tissue grafts on peri-implant tissue healing in the anterior or posterior sites in the maxilla or mandible.

The exclusion criteria established were animal or in vitro studies; any type of review; cohort studies; randomized clinical trials published before 2012; studies that included patients with uncontrolled systemic disorder; and editorials, abstracts in Congress, case reports, and case series.

### 2.3. Search Strategy and Data Extraction

Two calibrated researchers (E.M.R. and T.B.) executed the search strategy independently on the MedLine/PubMed and Cochrane Database platforms using the English language, human studies, and publications made since 2012 as search filters. Any disputes were resolved via confrontation and discussion between the two reviewers. The interrater reliability (Cohen’s kappa coefficient, κ) was performed to verify the degree of agreement between evaluators.

The bibliographic search consisted of a combination of MeSH terms and free-text words combined through Boolean Operators (AND or OR). The keywords used were the following: (1) PubMed/Medline: Dental implant [Mesh] OR dental implantation [Mesh] AND immediate implant placement [text word] AND graft [Mesh] AND bone [Mesh] OR bone graft [text word] OR buccal gap [text word]; filters: RCT-studies; 10 years studies; Human studies; English studies. (2) Cochrane: Dental Implant [Mesh] OR dental implantation [Mesh] AND immediate implant placement [text word] AND graft [Mesh] AND bone [Mesh] OR bone graft [text word] OR buccal gap [text word]; filter: None.

Data were extracted based on the general study characteristics, population characteristics, graft, and implant technique characteristics. Any discrepancy was solved with discussion and collaboration. The data were collected in predefined tables: general information, including study design, year of publication, number of patients, and patient information; information related to implant surgery and type of graft, including number of implants, implant location in the mouth, type of graft for both groups, follow-up, and follow-up intervals; information related to the surgical protocol; information related with the studies outcome variables; main outcomes: Pink Esthetic Score (PES), midfacial mucosa height, marginal bone loss (MBL), papilla index (PI), linear buccal change, and volumetric change; and secondary outcomes, including bleeding on probing (BoP) and plaque index.

### 2.4. Quality Assessment and Risk of Bias

The quality of the study was independently assessed by two reviewers (T.B. and E.M.R.). The risk of bias for RCTs was performed by using a revised Cochrane risk-of-bias tool for randomized trials (RoB2) [20]. The included parameters addressed with the tool (RoB2) were the following: the randomization process, deviation from intended interventions, missing outcome data, outcome measure, and selection of reported outcomes. If all parameters were filled with low risk (green) or up until there were two unclear (yellow), the overall result was Low Risk of Bias (green). For results with only one high risk (red) and up to two unclear (yellow), the result was Moderate Risk of Bias. Whereas, if filled with 2 or more High Risk (red) and/or more than 2 unclear risks (yellow), the overall result was High Risk of Bias.

A meta-analysis gathered the studies according to the similar analysis performed. A forest plot was developed using the random effect model to evaluate the effect size measures of standardized mean differences (95% confidence interval). Heterogeneity analysis was performed using Cochran’s Q test and Higgins’ I^2^, to verify if the existence of heterogeneity was the manifestation of differences between studies in relation to effect estimation. Percentages for I^2^ of 0–40%, 41–75%, and 76–100% of the mean were considered, respectively, as low, medium, and high heterogeneity. All statistical analyses were performed using the software Review Manager (v. 5.4).

## 3. Results

**Figure 1:**
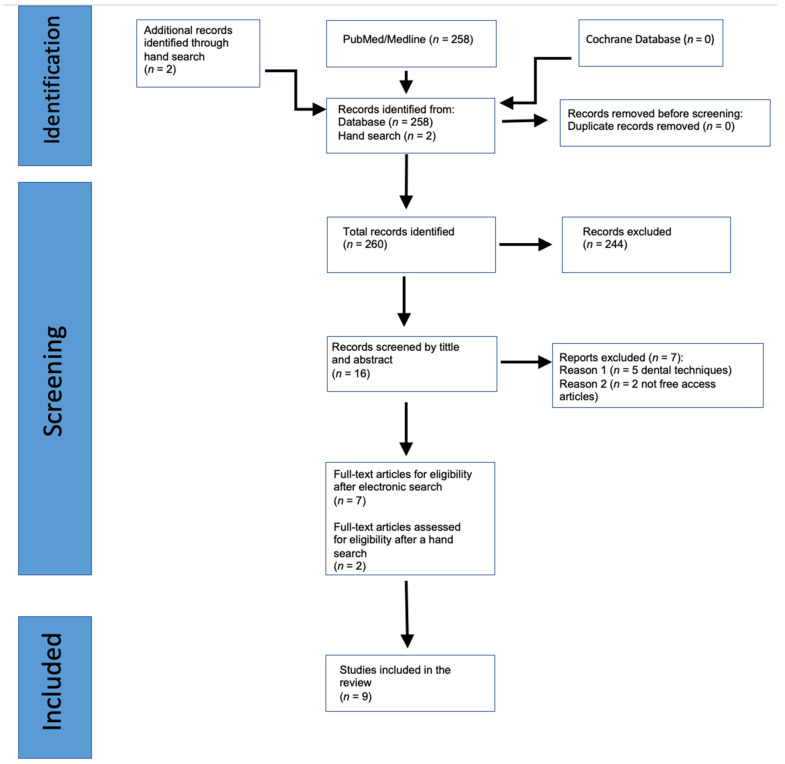
The flow diagram for the selection process is according to the PRISMA report (Preferred Reporting Items for Systematic Reviews and Meta-Analyses).

The initial electronic search identified 258 articles. Duplicate and triplicate articles were removed. The titles and abstracts of the potentially eligible articles concerning hard and soft tissue grafts in immediate implant placement (IIP) were carefully reviewed for eligibility. Two hundred forty-four publications were excluded, resulting in 14 articles (κ = 0.83). They were added to two hand-searched items, resulting in 16 articles chosen by title and abstract. The reasons for exclusion were studies not specific to dentistry (involved bone grafts in general surgery). Finally, the remaining 16 articles were examined via full-text evaluation. Nine articles were excluded (κ = 0.98). The reasons for the exclusion were as follows: (1) dental techniques did not meet the chosen criteria for the study and (2) inability to access the article. Finally, nine articles were included in the study [21,22,23,24,25,26,27,28,29]. The flow chart of the screening process is shown in Figure 1.

### 3.1. Study Characteristics

The characteristics of the included studies are described in Table 1, Table 2, Table 3, Table 4 and Table 5. Nine randomized clinical trials were analyzed with a total of 403 patients and 425 implants that we divided into three groups: bone graft (75 patients and 75 implants inserted), bone graft and membrane (213 patients and 235 implants inserted), and without bone graft (115 patients and 115 implants inserted). Regarding gender (Table 1), data were extracted only from seven out of the nine articles, totaling 156 men and 175 women [21,22,23,24,25,27,28].

The implant sites are detailed in Table 2. In three studies, implants were placed in the posterior area [24,26,27], whereas, in two RCTs, implants were placed in the anterior area, including premolars [25,28]. Regarding the arch, one RCT studied implants placed in the mandible (number of implants = 43) [27]; on the other hand, three articles placed implants in the maxilla (numbers of implants = 186) [24,25,28]. For the other included studies, it was not possible to obtain this information because of the lack of information.

Current smoking habit was considered as an exclusion criterion in six studies [21,22,23,25,28,29]. Regarding periodontal status, untreated/uncontrolled periodontal disease was an exclusion criterion in three studies [23,27,28]. No adverse effects were reported related to smoking or periodontal disease. For the alveolar bone condition, an intact buccal bone wall was considered as an inclusion criterion in four articles [22,23,25,28].

### 3.2. Characteristics and Results of Interventions (Table 4)

#### 3.2.1. Bone Grafting versus Extractive Technique without Bone Grafting

Four studies [21,22,23,24] compared the use of a bone graft and various extraction techniques without a bone graft.

#### 3.2.2. Alloplastic Graft with Membrane versus Extraction Technique (Naji et al., 2021 [22])

The authors reported a 100% implant success rate at the sixth-month follow-up. They found a significant reduction in the buccal bone plate at the 6-month observation in the flap extraction group without a graft, compared with the flap and flapless extraction group with a graft. No significant differences between the flap extraction with graft and flapless groups were found: the group with a graft presented changes of −0.37 ± 0.09 mm, the flap group −0.91 ± 0.54 mm, and the flapless group −0.24 ± 0.11 mm.

#### 3.2.3. Xenograft versus Socket Shield Technique (Atef et al., 2021 [23])

The authors reported a 100% implant success rate at the 12-month follow-up. They did not find a significant difference in terms of the total PES at 12 months. They did find, however, a statistically significant difference in MFR at 12 months: the socket shield group had an apical gingival migration of −0.45 (±0.75) mm compared with −0.466 (±0.58) mm in the xenograft group (*p* = 0.017). They also computed a significant difference in the horizontal dimensional change of the buccal bone at 6 months: the socket shield group 0.29 ± 0.34 mm and the xenograft group 1.45 ± 0.72 mm (*p* = 0.002). At the vertical bone level, the authors presented marginal bone changes of 0.36 ± 0.62 mm in the socket shield group and 1.71 ± 1.02 mm in the xenograft group (*p* = 0.008).

#### 3.2.4. Xenograft with Autogenous Graft and Membrane + VST Technique versus VST Technique without Grafting (Elaskary et al., 2022 [21])

The authors reported a 100% survival rate of the inserted implants. They found significant differences in terms of buccal bone thickness at the midpoint and apical level of the implant, but not at the crestal level, in favor of the VST technique with xenograft + autograft graft 1 year after implant insertion. The group treated with a VST technique with a graft presented middle bone mean values of 2.95 ± 0.97 mm and 3.75 ± 1.30 mm at the apical area, while the group treated without a graft showed a bone thickness of 1.82 ± 0.64 mm at the middle area and 2.03 ± 0.81 mm apically (*p* = 0.003 and *p* = 0.002, respectively). Similarly, the study identified a significant difference in the overall buccal alveolar ridge thickness level in favor of the grafted group at 1 year after surgery. An average bone increase of 2.95 ± 0.97 mm was noticed in this group, whereas in the non-graft group, only 1.98 ± 0.56 mm was computed (*p* = 0.003).

#### 3.2.5. Xenograft with Membrane versus Extraction (Mastrangelo et al., 2018 [24])

The authors reported a 99.1% implant success rate at the 1-year follow-up and 98.3% 3 years after implant placement. They found no significant association between the two groups regarding BoP measurements at 1 year and mucositis presence at the 3-year implant follow-up. On the other hand, the authors found a significant difference in distal and mesial bone levels at the third-year implant evaluation but not between the two groups. Similarly, they found significant PD differences at this point but not between the two groups. Also, the authors identified a statistically significant difference between the two groups in terms of PES score in favor of the graft-treated group: 9.7 ± 2.023 and 8.14 ± 1.895, respectively.

### 3.3. Different Types of Bone Grafting and/or Different Surgical Techniques

Five studies [25,26,27,28,29] compared different types of bone grafts associated with different surgical techniques.

#### 3.3.1. Xenograft with Dual-Zone Technique versus Xenograft (Wanis et al., 2022 [25])

The authors reported the failure of three implants (one in the test group and two in the control group). No significant differences in PES at 6 and 12 months were reported between the two groups. Similarly, they found no significant differences between the two groups in buccal bone changes, gingival recession, vestibular gingival thickness, and keratinized tissue.

#### 3.3.2. Xenograft versus Autogenous Graft (Noelken et al., 2020 [26]; Li et al., 2018 [27])

Noelken et al. [28] reported the failure of one implant from the xenograft group. They found no significant differences in implant survival, interproximal bone, buccal, and PD levels between the two groups at the 3-year follow-up period. Nevertheless, they found a statistically significant difference in favor of the xenograft with respect to buccal ridge thickness variation at 1 mm depth: the autogenous bone group decreased from 9.65 to 9.56 mm (−0.08 mm/−0.9%) while the xenograft group increased from 9.91 to 10.63 mm (+0.72 mm/+7.3%).

Li et al. [29] reported the failure of two implants but did not mention which group they belonged to. The authors found no significant difference in the ISQ of implant stability and marginal bone resorption between the two groups at 1 year. According to the authors, particulate derived from granulation of the extracted tooth appears to be a viable alternative for the GBR technique in immediate implant placement.

#### 3.3.3. Xenograft with Autogenous Graft and Connective Tissue Graft (CTG) versus Xenograft with Autogenous Graft (Van Nimwegen et al., 2018 [28])

The authors reported the failure of two implants (one per group), with a 1-year survival rate of 96.7%. Tissue volume loss was found in both groups, but no significant difference was identified between the two groups. However, a statistically significant difference was identified at the buccal mucosa level at 1 year in favor of the xenograft + autograft + CTG treated group. The group with the soft tissue graft presented a mean increase of +0.20 ± 0.70 mm compared with a mean loss of −0.48 ± 1.13 mm in the group treated without using the CTG. This may be translated to a nonsignificant difference in terms of soft tissue volume reduction in the post-extraction phase but to greater tissue stability when using a connective tissue membrane. No significant difference was found between the two groups in total PES, PD, and Plaque Index at 1-year follow-up.

#### 3.3.4. Xenograft versus Xenograft with Collagen Matrix versus Xenograft with Autogenous CTG (Frizzera et al., 2018 [29])

The authors reported a 100% success rate of the inserted implants. The total PES found no significant differences between the groups at 6 months and 1 year. Nevertheless, they stated that one of the PES scores, the alveolar process, was significantly better in the group treated without a membrane or CTG. In contrast, the PES score regarding gingival recession favors the group treated with graft and CTG. The authors found no significant differences in bone thickness and bone resorption between the three groups at 6 months and 1 year after implant insertion.

### 3.4. Clinical Outcomes

#### 3.4.1. Mid-Facial Mucosa Level at 12 Months

Gingival recession at 1 year was calculated in the work of Frizzera et al. [29], Van Nimwegen et al. [28], and Wanis et al. [25]. In Frizzera et al.’s [29] and Van Nimwegen et al.’s [28] studies, the authors found better results when a connective tissue membrane is combined with the xenogeneic graft. Wanis et al.’s [25] study reported better results with a combination of the dual-zone technique with a xenogeneic graft.

#### 3.4.2. Total PES at 12 Months

Total PES at 1 year was considered by Frizzera et al. [29], Wanis et al. [25], and Van Nimwegen et al. [28]. The authors found no significant difference in using a xenogeneic graft with or without a membrane.

#### 3.4.3. Facial Bone Thickness at 12 Months

Facial bone thickness was studied by Naji et al. [22] and Elaskary et al. [21]. The authors found better yields in the graft-treated and flapless groups.

### 3.5. Quality Assessment and Risk of Bias

The risk of bias was evaluated by using a revised Cochrane risk-of-bias tool for randomized trials (RoB2). Four studies had low risk of bias, four had moderate risk of bias, and one had high risk of bias (Table 5).

### 3.6. Meta-Analysis

**Figure 2:**
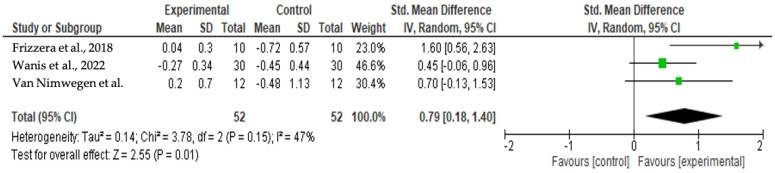
Results of the meta-analysis for the mid-facial mucosa level (12 months) [25,28,29].

**Figure 3:**
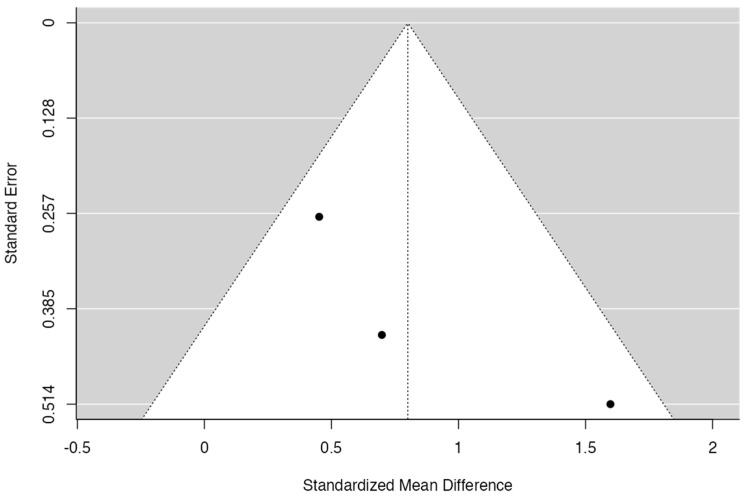
Funnel plot for the mid-facial mucosa level (12 months).

Given the results of Figure 2 and Figure 3, Cochran’s Q had a *p*-value of 0.15 and I^2^ = 46%; then, it was possible to verify a medium level of heterogeneity for the mid-facial mucosa level analysis. The forest plot shows that the meta-analysis effect of 0.79 (95% CI [0.18; 1.40]) was statistically significant (*p* = 0.01). There was a tendency to favor the experimental group in all articles included for this analysis.

**Figure 4:**
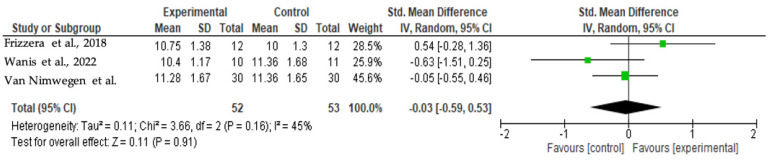
Results of meta-analysis for the total PES (12 months) [25,28,29].

**Figure 5:**
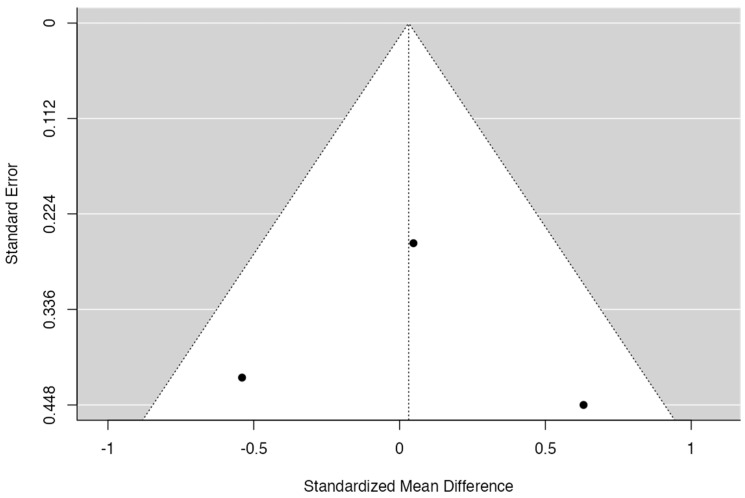
Funnel plot for the total PES (12 months).

Considering the results of Figure 4 and Figure 5, Cochran’s Q had a *p*-value of 0.16 and I^2^ = 45%; thus, it was possible to verify a medium level of heterogeneity among studies regarding total PES. The forest plot shows no significant differences between studies (*p* = 0.91), although Frizzera et al. [29] presented favoring toward the experimental group, and Wanis et al. [25] toward the control group.

**Figure 6:**
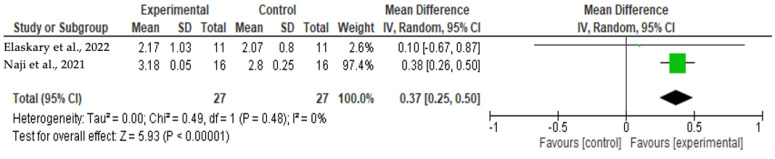
Results of meta-analysis for facial bone thickness (12 months) [21,22].

**Figure 7:**
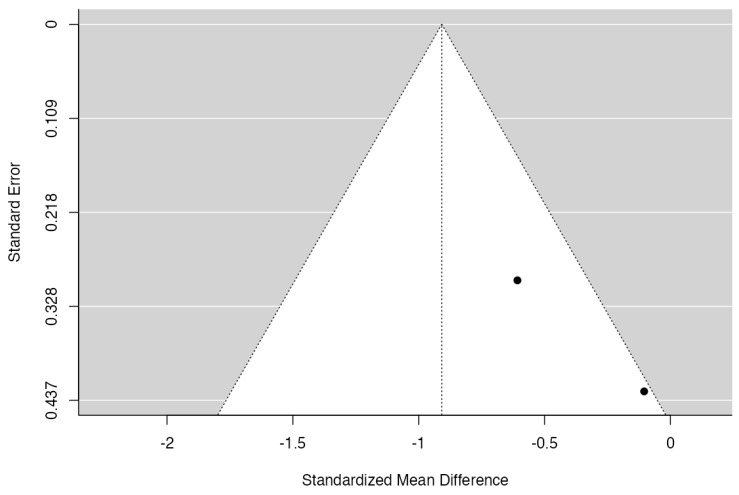
Funnel plot for facial bone thickness (12 months).

Figure 6 and Figure 7 show homogeneity (I^2^ = 0%) among the facial bone thickness analysis studies. The Cochran’s Q analysis had a *p*-value of 0.48, presenting no statistical differences between the studies evaluated, with both favoring the experimental group. Naji et al.’s [22] study had a higher weight (97.4%) in this analysis. The forest plot shows that the meta-analysis effect of 0.37 (95% CI [0.25;0.50]) was statistically significant (*p* < 0.00001).

## 4. Discussion

The aim of this systematic study was to evaluate the efficacy of hard and/or soft tissue grafts associated with type-1 implants (IIP) on healing and treatment outcomes to provide a more predictable result. Our study included only RCTs that evaluated IIP with at least one group using hard and/or soft tissue grafts.

Buser et al. [9,30] identified some factors that may increase the risk of esthetic predictability in IIP cases: (1) thin gingival biotype, (2) thin buccal bone wall, and (3) implant surgical procedure by itself. Also, Buser et al. [30] showed a greater risk of having a 1 mm gingival recession and greater variability in esthetic results when using the immediate implant technique. This was also stated by Bakkali et al. [31], who argued that there is less esthetic predictability using this approach. On the other hand, Siqueira et al. [32] showed a case report of IIP combined with CTG, demineralized bovine bone mineral with 10% collagen, and immediate provisional crown adjusted intra- and extra-orally (establishing the ideal critical and subcritical contour), with high tissue stability after 4 years. Among the included studies, only two considered and used the CTG associated with IIP [28,29], with improved clinical results compared to the control group. Even though it has been shown that a membrane promotes bone neoformation by positively influencing bone remodeling as a scaffold between implant and bone [33], two studies [22,24] that considered the utilization of collagen membranes did not have significant results.

Borges et al. [34] pointed out that a buccal bone ridge thinner than 1 mm is the primary determinant of buccal tissue reduction at 1-year post-treatment with immediate dental implants. The authors showed that pretreatment of this anatomic condition is important in cases where the individual is also diagnosed with a thin gingival biotype [34]. Due to this concern (the presence of buccal bone ridge thinner than 1 mm), several authors have advocated using bone grafts, which could significantly reduce peri-implant tissue reduction [34,35,36]. Moreover, bone grafts contributed to horizontal bone preservation and soft tissue stability at the midfacial aspect of immediate implants, which should be considered as an adjunct to IIP in clinical practice [7]. Elaskary et al. [21] reported improved clinical yield regarding the buccal bone ridge when the post-extraction gap was treated with a graft. The experimental group, treated with a VST technique and a graft, presented a facial bone thickness of 2.95 ± 0.97 mm at the mid-level and 3.75 ± 1.30 mm at the apical level of the alveolar ridge, in contrast with the control group (without a graft) which presented values for the buccal bone of 1.82 ± 0.64 mm and 2.03 ± 0.81 mm, respectively. Therefore, the results must be carefully interpreted due to the limited number of studies included and the heterogeneity found.

It is essential to highlight that if the remnant post-extraction buccal gap size exceeds 1.5 mm, it might achieve incomplete bone regeneration if left to spontaneous heal [37]. This fact agrees with Naji et al.’s results [22], which indicated that the group with the flap technique and a graft had better buccal bone maintenance (buccal bone changes of −0.37 ± 0.09 mm) compared to the group with a flap without a graft (buccal bone changes of −0.91 ± 0.54 mm). The same authors also advocated that, if possible, applying the flapless technique can substantially improve the tissue healing performance compared to the use of a flap, preventing or reducing the buccal bone resorption; this can be explained by a reduction in the breaking of the local vascularization; the blood perfusion for the buccal bone ridge comes essentially from the periodontal ligaments, periosteum, and bone marrow [38]. In addition, they reported better results using a membrane than first intention closure, which agrees with a systematic review [31] that supported using bone grafts to reduce the buccal bone wall resorption after tooth extraction. However, the results presented in our study must be carefully interpreted because of the small sample size present in some of the included articles and the moderate/high risk of bias observed.

Clearly, esthetic factors are influenced by the presence or absence of a substantial buccal bone volume. Without a sufficient buccal bone plate, marginal bone resorption will result in volumetric alteration of the peri-implant soft tissue [39]. This theory entirely agrees with Guarnieri et al.’s [10] arguments, which include the believe that bone loss and the likelihood of soft tissue volume reduction are directly proportional: greater bone loss will likely cause a gingival recession. Fernandes and collaborators [40] stated that the predictability of these outcomes in IIP is related to correct patient selection criteria. However, they also argued that there is a lack of objectivity in the methods used in studies to evaluate esthetic outcomes, which often depend on the observer, reducing reproducibility among different observers and studies [40]. Wanis et al.’s study [25] agrees with these findings; they claimed that both groups revealed a PES value between 10 and 12, which is considered a good result. In contrast, Abd-Elrahman et al. [41], who observed similar groups to those within Wanis et al.’s [25] study, but without the use of bone grafts, reported a significantly lower PES at 6-month follow-up (8.85 ± 1.81). Wanis et al. [25] reported that using a surgical technique without a graft at the post-extraction buccal gap was responsible for the reduced total PES value; they stated that no real prevention of the buccal bone loss was observed in either of the groups at 6 and 12 months after implant insertion: −0.88 mm ± 0.41 in the dual-zone technique group and −1.08 mm ± 0.28 in the group with the graft alone.

The literature suggests some variability in terms of the mean MBL associated with type-1 implants with various surgical techniques and grafts: Siqueira et al. [42] reported a −0.66 ± 0.38 mm mean of marginal bone reduction while Pardal-Pelaez et al. [43] reported −0.42 ± 0.78 mm and Mazzocchi et al. [44] reported −0.48 ± 0.76 mm. These slight changes could be due to the different assessment techniques, which are unequal and operator-dependent [25]. Sanz et al. [45] had previously reported a significant reduction in MBL when IIP was associated with using a bone graft: −1.1 mm (graft group) compared to −1.6 mm in the non-graft group. However, several authors consider the intact buccal bone plate and gingival biotype beyond other factors, such as the flapless technique and the distance between implant shoulder and cortical bone, as determinants for the buccal bone healing at IIP sites, regardless of the presence of large gaps and the use of different grafts [46]. Furthermore, Elaskary et al. [21] argued that the first six months after surgery is the most critical period concerning post-extraction bone resorption. This is also supported by Borges et al. [34] and Lops et al. [47].

In addition, the literature has limitations regarding studies on compromised post-extraction sockets [11]. Most studies report positive data for IIP techniques in fresh and intact sockets [29,48,49] but strongly question such outcomes when the technique is used in damaged/compromised post-extraction ones [50,51,52,53]. Elaskary et al. [21] argued that when faced with such eventuality, the clinician should always opt for bone regeneration with a membrane in conjunction with immediate implantation to achieve better esthetic and functional results. These authors reported better results when the xenograft is combined with a CTG (from the patient’s palate). They also reported a higher PES score regarding the level of marginal mucosal tissue [21]. This fact was also highlighted by Van Nimwegen et al. [28], who hypothesized the association between a xenograft and CTG might provide greater stability to peri-implant tissues, namely at the marginal mucosal level. Nevertheless, the authors concluded that using a CTG associated with a xenogeneic bone graft may not counteract the volumetric tissue changes after IIP. The data presented did not show any significant differences in terms of volumetric reduction of the soft tissue, but it had positive effects on peri-implant marginal mucosa stability one year after treatment: the test group presented an average increase of 0.20 ± 0.70 mm compared to an average loss of −0.48 ± 1.13 mm in the control group [28]. They also reported significantly higher marginal gingival PES results than those without connective tissue grafts, even though total PES values showed no significant statistical differences between the two groups. Then, the authors concluded that CTG should be associated with IIP to reduce the possibility of asymmetry between peri-implant soft tissue and adjacent teeth [28].

The limitations of this review can be assigned to different items. We included only articles published in English; given the relatively recent nature of these techniques, the literature is still sparse, and we only included nine RCTs, following the inclusion criteria initially established. In addition, one of the databases consulted had no search results. Also, for the selected articles, after careful independent analysis by the two independent researchers (EMR and TB), we found considerable variability in terms of the chosen variables included in the studies and evaluation techniques that were not always objective, repeatable, and comparable; some articles had a small sample size, the presence of heterogeneity was found, and moderate/high RoB was observed, which suggests a careful interpretation of the data obtained. These reasons explain why we were able to conduct a meta-analysis of a low number of variables, selecting a total of five articles, which were divided among three comparable parameters. Moreover, because of the variability of the evaluation techniques and variables, it was necessary to work on interpretation and equivalence to summarize the data into clear and universal parameters that could be used to group the highest number of publications.

## 5. Conclusions

Within the limitations of this study, it was possible to conclude that using bone and soft tissue grafting techniques associated with IIP, even though they are not fundamental, were a valuable resource to prevent significant tissue reduction, reaching greater bone stability and higher levels in the Pink Esthetic Score (PES) and Visual Analogue Score (VAS). Results also may depend on the professional’s surgical and clinical ability/experience. In addition, the use of CTG combined with a xenogeneic bone graft brought advantages to the mid-facial mucosa position around immediate implants. It is important to state that standardized clinical assessment techniques and objective criteria are needed for comparisons in future studies.

## Figures and Tables

**Table 1:** Demographic information.

Author	Year	N	Age Range	Gender (Male/Female)
**Elaskary et al.** [21]	2022	22	Mean 45	M: 8 F: 14
		Group I (intervention): 11	Group I: 44.63	Group I: M: 5 (45.5%) F: 6 (54.5%)
		Group II (control): 11	Group II: 45.81	Group II: M: 3 (27.3%) F: 8 (72.7%)
**Naji et al.** [22]	2021	48	28–55	F: 30 M: 18
		Group I (intervention): 16	Group I: 40.2	Group I: M: 5 (31.25%) F: 11 (68.75%)
		Group II (control I): 16	Group II: 43.3	Group II: M: 7 (43.75%) F: 9 (56.25%)
		Group III (control II): 16	Group III: 41.1	Group III: M: 6 (37.5%) F: 10 (62.5%)
**Atef et al.** [23]	2021	42	>18	M: 25% F: 75%
		Intervention Group: 21	mean 36	Test group: M: 5 (25%) F: 15 (75%)
		Control group: 21		Control Group: M: 5 (25%) F: 15 (75%)
**Mastrangelo et al.** [24]	2018	102	18–72	M: 63 F: 39
		Group A (intervention): 51	Mean 44	Group A: M: 31 (60.7%) F: 20 (39.2%)
		Group B (control): 51		Group B: M: 32 (62.7%) F: 19 (37.2%)
**Wanis et al.** [25]	2022	24	20–45	M: 7 F: 17
		DZ Group (intervention): 12	DZ Group: 34.27	DZ Group: M: 4 (36%) F: 7 (63.4%)
		BCG Group (control): 12	BCG Group: 30.30	BCG Group: M: 3 (30%) F: 7 (70%)
**Noelken et al.** [26]	2020	50	23–73	M: 18 F: 32
		AB Group (control): 25	Mean 47	
		BBGM Group (intervention): 25		
**Li et al.** [27]	2018	40	20–60	M: 24 F: 16
		DDM Group (control): 20	DDM Group: 36.6	DDM Group: M: 11 (57.8%) F: 8 (42.10%)
		BIO Group (intervention): 20	BIO Group: 34.9	BIO Group: M: 11 (43.75%) F: 8 (56.25%)
**van Nimwegen et al.** [28]	2018	60	19–82	M: 28 F: 32
		Intervention Group (CTG): 30	Test Group: 19.5–67.84 (mean 45.5)	Test Group: M: 13 (43.3%) F: 17 (56.5%)
		Control Group: 30	Control Group: 20.9–82.2 (mean 47.8)	Control Group: M: 15 (50%) F: 15 (50%)
**Frizzera et al.** [29]	2018	24	23–65	M: 7 F: 17
		CTL Group (control): 8		
		CM Group (Intervention I): 8		
		CTG Group (Intervention II): 8		

M—male; F—female; AB—autogenous bone; BBGM—biphasic bone graft material; DDM—demineralized dentin matrix; BIO—Bio-Oss; CTL—control; CM—collagen matrix; CTG—connective tissue graft; DZ—dual-zone therapeutic concept; BCG—buccal bone crest with immediate temporization.

**Table 2:** Graft and implant information.

Author	Follow-Up	Intervals	Type of Graft	Implant Placement (Site)	Implant (*n*)	Outcome
**Elaskary et al.** [21]	1 year	T0: baseline preextraction T1: 1 year	Group I: particulate bone graft 75% autogenous bone chips harvested form local surgical sites and 25% deproteinized bovine bone mineral (DBBM) of bovine origin (MinerOss X Cortical Particle Size, 500–1000 microns) (Biohorizons Implant Systems, Birmingham, Alabama, USA) GROUP II: no graft	Esthetic zone	22	Buccal bone thickness
**Naji et al.** [22]	6 months	T0: before extraction T1: immediately after implant placement T2: 6 months	GROUP I: alloplastic nanocrystalline calcium sulphate bone graft (Orthogen LLC, Springfield, New Jersey, USA) and an absorbable collagen membrane (Bioimplon GmbH, Gießen, Germany) GROUP II–III: without graft and membrane	Upper premolar tooth	52	CBCT bone examination Pain intensity
**Atef et al.** [23]	1 year	T0: casts before the extraction T1: CBCT immediately post placement of implant T2: CBCT after 6 months T3: photos, casts and patients satisfaction 12 months	Test group: without graft + collagen plug; Control group: with bovine cancellous xenograft (Tutobone, Tutogen Medical GmbH, Neunkirchen a. Brand, Germany) + collagen plug	26: premolar tooth 16: upper incisors and canine area	42	Peri-implant soft tissue PES Midfacial mucosa alteration Change in the buccal bone I-C (vertical) Change in the buccal bone I-OS (horizontal) Patient satisfaction
**Mastrangelo et al.** [24]	3 years	Radiographic and clinical periodontal assessment T0: 3 months T1: 1 year T2: 3 years	Group A: granular bone grafting was inserted (BioOss, Geistlich, Germany), which completely covered the pericardium membrane (Osteobiol Evolution, Tecnoss, Italy) Group B: no graft and barrier	Upper premolar: 36: 1.4 26: 1.5 30: 2.4 23: 2.5	115	Implants failure Marginal bone loss PES Pocket depth Biological complications
**Wanis et al.** [25]	1 year	T0: baseline T1: 6 months T2: 1 year	DZ Group—BCG Group: bone graft cortico-cancellous collagenated bone grafting material of porcine origin pre-hydrated and collagenated cortico-cancellous porcine bone, 250–1000 μm, Gen-Os^®^ (Osteobiol, Technoss Dental S.r.l.) DZ Group: dual technique zone BCG Group: flapless technique	6: upper central 5: upper lateral 1: canines 5: first premolar 4: second premolar	24	PES BBL: Buccal bone changes (horizontal) via probe MFR: The midfacial recession STT: The soft tissue thickness at 2–4–6 mm KTW: The keratinized tissue VAS for POS PS
**Noelken et al.** [26]	3 years	T0: baseline T1: placement implant T2: 1 year (*n* = 8 implants) T3: 2 years (*n* = 16 implants) T4: 3 years (*n* = 24 implants)	AB Group: autogenous bone grafts were harvested at the mandibular ramus by collecting bone particles with a disposable bone scraper (Micross, META). BBGM Group: a resorbable, biphasic, and anorganic graft material of plant origin derived from red algae (BBGM) (Symbios, Dentsply Sirona).	Molar of the maxilla and the mandible 34: mandibular implants 16: maxillary implants	50	Implant survival rate Marginal bone level changes Buccal bone level Buccal width of the alveolar crest Pocket depths Implant success rate Plaque index BoP
**Li et al.** [27]	18 months	Radiographic T0: baseline T1: 6 months T2: 18 months	DDM Group: autogenous DDM granules from the extracted tooth BIO Group: Bio-Oss (Geistlich Pharma AG, Wolhusen, Switzerland) cancellous granules	Lower premolar: 19 Lower molar: 25	45	ISQ Measurements of marginal bone resorption
**Van Nimwegen et al.** [28]	1 year	T0: preextraction clinical parameters, photos, and impression T1: 1-year, clinical parameters, photos, and impression	Test and Control Group: a 1:1 mixture of autogenous bone and anorganic bovine bone (Geistlich Bio-Oss^®^; Geistlich Pharma AG, Wolhusen, Switzerland) Test Group received connective tissue graft (CTG), which was harvested from the tuberosity region	Maxilla Incisor: 47 Maxilla Canine: 10 Maxilla Premolar: 3	60	Volumetric change: thickness Midfacial mucosa recession Gingival biotype Implant probing depths Plaque scores Bleeding scores Mucosa inflammation PES Patient satisfaction: VAS
**Frizzera et al.** [29]	12 months	T0: baseline T1: 6 months T2: 12 months	CTL Group: no soft tissue graft CM Group: graft of collagen matrix (Mucograft, Geistlich) CTG Group: CTG from palate The facial space was filled with bovine bone mineral containing 10% porcine collagen (Bio-Oss Collagen, Geistlich) placed between the membrane and the dental implant	13: 1.1/2.1 11: 1.2/2.2	24	MPR Implant success rate Papilla migration PES Soft tissue thickness Bone thickness

CBCT—Cone Beam Computed Tomography; CTL—control; CM—collagen matrix; CTG—connective tissue graft; BIO—Bio-Oss; AB—autogenous bone; BBGM—biphasic bone graft material; DDM—demineralized dentin; DZ—dual-zone therapeutic concept; BCG—buccal bone crest with immediate temporization; PES—Pink Esthetics Score; MPR—marginal peri-implant recession; VAS—visual analogue scale; ISQ—implant stability quotient; BoP—bleeding on probing; PS—patient satisfaction; POS—postoperative swelling.

**Table 3:** Surgical protocol.

Author	Surgical Protocol
Elaskary et al. [21]	Atraumatic tooth extraction and the VST protocol. Then, a cortical membrane shield was made of heterologous origin and introduced through the tunnel apically.
	Group I: using the graft
	Group II: not using the graft.
Naji et al. [22]	For group I and II a full thickness flap.
	The junction gap was filled.
	Group II was treated without bone graft or membrane.
	Group III healing was free.
Atef et al. [23]	Test Group: the socket shield technique.
	Control Group: atraumatic extraction following implant placement; the junction gap was filled with bovine cancellous xenograft.
	A piece of a collagen plug was placed to close the entrance of the extraction socket in both groups.
Mastrangelo et al. [24]	Tooth extraction with mucoperiosteal flap. The immediate implant was inserted.
	Group A: graft and barrier healing.
	Group B: no graft and barrier.
Wanis et al. [25]	A flapless minimally traumatic extraction technique. The immediate implants were inserted.
	DZ Group: the bone graft filled the junction gap to wall up to the free gingival margin.
	BCG Group: the bone graft filled the junction gap; the graft was packed just reaching the buccal bone crestal level.
Noelken et al. [26]	Atraumatic flapeless extraction technique. The immediate implants were inserted.
	The junction gap was filled with AB or BBGM graft.
	The graft was additionally covered with a platelet-rich fibrin (PRF) membrane.
Li et al. [27]	Tooth extraction with a mucoperiosteal flap. Immediate implant was inserted.
	The junction gap was filled with a graft and injectable PRF and membrane barrier for healing.
Van Nimwegen et al. [28]	Atraumatic flapless extraction technique. The junction gap was filled with xenograft inorganic bovine before the insertion of the immediate implant. In the test group, a connective autogenous graft was utilized.
Frizzera et al. [29]	Atraumatic tooth extraction and implant placement with immediate loading of a provisional crown. A bovine graft was utilized in every group.
	CTL Group: no soft tissue graft.
	CM Group: graft of collagen matrix.
	CTG Group: tissue autogenous graft from the palate

CTL—control; CM—collagen matrix; CTG—connective tissue graft; AB—autogenous bone; BBGM—biphasic bone graft material; DZ—dual-zone therapeutic concept; BCG—buccal bone crest with immediate temporization; VST—Vestibular Socket Therapy; PRF—platelet-rich fibrin.

**Table 4:** Clinical outcomes of selected studies.

Author	Outcome		
**Elaskary et al.** [21]	**Comparison of the overall bone thickness**:		
	**Baseline**		
	Group I: 1.45 ± 0.92 mm	Group II: 0.79 ± 0.49 mm	
	**12 months**		
	Group I: 2.95 ± 0.97 mm	Group II: 1.98 ± 0.56 mm	
**Naji et al.** [22]	**CBCT Bone examinations**		
	**Mean value of the buccal bone plate thickness + horizontal gap width at T1 was:**		
	**Group I**:	**Group II:**	**Group III:**
	**T1:** 3.56 ± 0.10 mm	**T1:** 3.71 ± 0.57 mm	**T1:** 3.43 ± 0.33 mm
	**T2:** 3.18 ± 0.05 mm	**T2:** 2.80 ± 0.25 mm	**T2:** 3.19 ± 0.28 mm
	**T2–T1 =** −0.37 ± 0.09 mm	**T2–T1 =** −0.91 ± 0.54 mm	**T2–T1 =** −0.24 ± 0.11 mm
	**PAIN INTENSITY**		
	**Group I**:	**Group II:**	**Group III:**
	5.14 ± 0.69	3.71 ± 0.76	0.71 ± 0.49
**Atef et al.** [23]	**Mid-facial mucosal alteration**		
	**Control group** **(xenograft)**	**Test group** **(socket shield)**	
	−0.466 ± 0.58 mm	0.45 ± 0.75 mm	
	**Radiographic outcomes**		
	**The change in the buccal** **(I-C):**		
	**Control group**	**Test group**	
	1.71 ± 1.02 mm	0.36 ± 0.62 mm	
	**The change in the buccal** **(I-OS):**		
	**Control group**	**Test group**	
	1.45 ± 0.72 mm	0.29 ± 0.34 mm	
	**Patient satisfaction vas score** **(12 months):**		
	**Control group**	**Test group**	
	9.25 (±0.70)	9.37 (±0.80)	
	**PES**		
	**Control group**	**Test group**	
	11.86 ± 0.35	12.12 ± 0.64	
**Mastrangelo et al.** [24]	**Implants failure**:		
	Group A: 1	Group B: 1	
	**Marginal bone level**		
	**T0–T2**		
	Group A: −0.25 ± 0.362 mm	Group B: −0.28 ± 0.3 mm	
	**PES**		
	Group A: 8.14	Group B: 9.7	
	**Probing depth**		
	**T0–T2**		
	Group A: 1.69 ± 1.34 mm	Group B: 1.4 ± 1.61 mm	
	**Biological complications like fistulas, mucositis, and periimplantitis**: 58 patients		
**Wanis et al.** [25]	Two implants failed osteointegration after 2 months post-surgery (one from each group).		
	**PES**		
	**Baseline**:	**6 months**	**12 months**:
	DZ Group: 10.82 (±1.54)	DZ Group: 11.09 (±1.58)	DZ Group: 11.36 (±1.69)
	BCG Group: 10.10 (±1.20)	BCG Group: 10.40 (±1.17)	BCG Group: 10.80 (±1.55)
	BBL (at 0 mm):		
	**6 months**	**12 months**:	
	DZ Group: 0.67 (±0.43) mm	DZ Group: 0.88 (±0.41) mm	
	BCG Group: 0.84 (±0.26) mm	BCG Group: 1.08 (±0.28) mm	
	BBL (at 2 mm):		
	**6 months**	**12 months**:	
	DZ Group: 0.59 (±0.32) mm	DZ Group: 0.82 (±0.32) mm	
	BCG Group: 0.51 (±0.27) mm	BCG Group: 0.79 (±0.30) mm	
**Noelken et al.** [26]	**Implant survival rate**		
	**AB Group**:	**BBGM Group**:	
	100%	96%	
	**Mean interproximal bone level**		
	**AB Group T1**:	**BBGM Group T1**:	
	Min: −13.2 mm	Min: −11.86 mm	
	Max: −2.19 mm	Max: −3.80 mm	
	Mean: −7.36 mm	Mean: −7.6 mm	
	**AB Group final**:	**BBGM Group final**:	
	Min: −0.87 mm	Min: −1.83 mm	
	Max: −1.85 mm	Max: 1.93 mm	
	Mean: 0.38 ± 0.78 mm	Mean: 0.1 ± 0.78 mm	
	**Mean vertical distance from implant shoulder to the bottom of the buccal bone defect**		
	**AB Group T1**:	**BBGM Group T1**:	
	−7.18 ± 3.43 mm	T1: −6.59 ± 2.65 mm	
**Li et al.** [27]	**ISQ**		
	**DDM Group**		
	**T0**:	**T1**:	**T3**:
	53.6 ± 11.9 mm	77.6 ± 7.9	79.5 ± 6.0 mm
	**BIO Group**		
	**T0**:	**T1**:	**T3**:
	54.1 ± 13.0 mm	78.1 ± 4.2	80.2 ± 4.3 mm
	**Marginal bone resorption around implant**		
	**DDM Group**		
	**T1**:	**T2**:	
	1.7 ± 0.3 mm	1.9 ± 0.6 mm	
	**BIO Group**		
	**T1**:	**T2**:	
	1.8 ± 0.1 mm	2.0 ± 0.5 mm	
**Van Nimwegen et al.** [28]	**Volumetric change**		
	**A. Thickness (T0–final):**		
	**Control group**:	**Test group**:	
	−0.49 ± 0.54 mm	0.68 ± 0.59 mm	
	**B. Mid-facial mucosa (T0–final):**		
	**Control group**:	**Test group**:	
	−0.48 ± 1.13 mm	0.20 ± 0.70 mm	
	**PD at 1 year**		
	**Control group**:	**Test group**:	
	2.44 ± 1.19 mm	2.28 ± 0.79 mm	
	**PES**		
	**Control group**:	**Test group**:	
	11.36 ± 1.65	11.28 ± 1.67	
**Frizzera et al.** [29]	**PES**		
	**Baseline**:	**12 months**:	
	(CTL Group) 10.75 (±2.05)mm	(CTL Group) 9.87 (±1.64) mm	
	(CM Group) 10.63(±1.84) mm	(CM Group) 10 (±1.3) mm	
	(CTG Group) 9.37(±1.9) mm	(CTG Group) 10.75 (±1.38) mm	
	**MP (mesial papilla migration)**		
	**6 months**:	**12 months**:	
	(CTL Group) 0.64 (±0.41) mm	(CTL Group) 0.36 (±0.7) mm	
	(CM Group) 0.39 (±0.45) mm	(CM Group) 0.41 (±0.47) mm	
	(CTG Group) 0.53(±0.28) mm	(CTG Group) 0.56 (±0.57) mm	
	**DP** **(distal papilla migration)**		
	**6 months**:	**12 months**:	
	(CTL Group) 0.69 (±0.62) mm	(CTL Group) 0.74 (±0.68) mm	
	(CM Group) 0.64 (±0.80) mm	(CM Group) 0.52 (±0.67) mm	
	(CTG Group) 0.44 (±0.79) mm	(CTG Group) 0.47 (±0.53) mm	
	**MPR** **(marginal peri-implant recession)**		
	**6 months**:	**12 months**:	
	(CTL Group) 0.41 (±0.40) mm	(CTL Group) 0.72 (±0.57) mm	
	(CM Group) 0.14 (±0.37) mm	(CM Group) 0.42 (±0.60) mm	
	(CTG Group) −0.41 (±0.75) mm	(CTG Group) −0.04 (±0.3) mm	

CBCT—Cone Beam Computed Tomography; CTL—control; CM—collagen matrix; CTG—connective tissue graft; BIO—Bio-Oss; AB—autogenous bone; BBGM—biphasic bone graft material; DDM—demineralized dentin; DZ—dual-zone therapeutic concept; BCG—buccal bone crest with immediate temporization; MPR—marginal peri-implant recession; BBL—buccal bone loss.

**Table 5:** Overall risk-of-bias assessment using the Cochrane risk-of-bias 2 (RoB2) tool.

Articles	Randomization Process	Deviations fromThe Intended Interventions	Missing Outcome Data	Measurement of the Outcome	Selection of the Reported Result	Overall
Naji et al. [22]	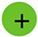	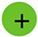	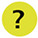	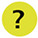	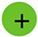	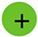
Atef er al. [23]	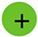	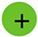	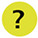	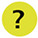	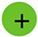	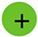
Wanis et al. [25]	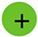	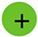	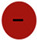	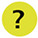	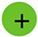	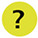
Noelken et al. [26]	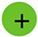	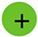	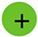	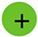	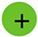	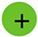
van Nimwegen et al. [28]	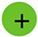	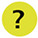	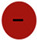	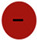	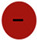	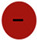
Elaskary et al. [21]	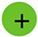	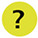	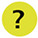	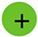	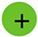	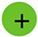
Mastrangelo et al. [24]	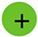	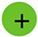	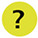	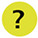	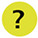	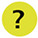
Li et al. [27]	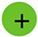	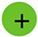	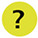	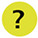	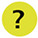	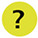
Frizzera et al. [29]	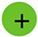	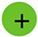	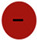	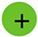	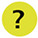	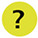

## Data Availability

All data obtained were included in this article.
